# Predictors of Receiving Help with Banking Among Older Adults With Probable Dementia

**DOI:** 10.1093/geroni/igaf122.3999

**Published:** 2025-12-31

**Authors:** Alina Kung, Claire Ankuda

**Affiliations:** Icahn School of Medicine at Mount Sinai, New York, New York, United States; Icahn School of Medicine at Mount Sinai, New York, New York, United States

## Abstract

**Background:**

Receiving help with financial matters has the potential to protect older adults with cognitive impairment from adverse economic consequences. Little is known on who receives help with banking among these older adults.

**Methods:**

We used data from the National Health and Aging Trends Study to identify a cohort of older adults (aged 65 years and older) with probable dementia (n = 4048) (hereafter, OAPD). We used the first survey where the participant reported probable dementia, and excluded observations with incomplete data (n = 851). We used multivariate logistic regression to identify sociodemographic, household, health, and functional predictors associated with receiving help with banking in the last month.

**Results:**

Only about half of OAPD received help with banking (n = 1656, 52.7%). In adjusted analyses, being older (aOR 2.42 for age group >90 versus age group 65-69), female (aOR 1.33), not living with a spouse (aOR 1.69), on Medicaid (aOR 1.33), in poorer self-reported health (aOR 2.29 for poor health versus excellent health) or having a proxy answer the interview (aOR 2.92) were associated with receiving help with banking in the last month.

**Conclusion:**

About half of OAPD do not receive help with banking, indicating a potential risk and unmet need. OAPD who are younger, male, living with a spouse, and not on Medicaid are more likely to not receive help with banking. Future work should explore which OAPD are at greatest risk of adverse economic and health consequences as a result of not having received helped with banking.

